# The Source of Spontaneous Activity in the Main Olfactory Bulb of the Rat

**DOI:** 10.1371/journal.pone.0023990

**Published:** 2011-08-30

**Authors:** Josif Stakic, Jessica M. Suchanek, Geoffrey P. Ziegler, Edwin R. Griff

**Affiliations:** Department of Biological Sciences, University of Cincinnati, Cincinnati, Ohio, United States of America; Center for Genomic Regulation, Spain

## Abstract

**Introduction:**

*In vivo*, most neurons in the main olfactory bulb exhibit robust spontaneous activity. This paper tests the hypothesis that spontaneous activity in olfactory receptor neurons drives much of the spontaneous activity in mitral and tufted cells via excitatory synapses.

**Methods:**

Single units were recorded in vivo from the main olfactory bulb of a rat before, during, and after application of lidocaine to the olfactory nerve. The effect of lidocaine on the conduction of action potentials from the olfactory epithelium to the olfactory bulb was assessed by electrically stimulating the olfactory nerve rostral to the application site and monitoring the field potential evoked in the bulb.

**Results:**

Lidocaine caused a significant decrease in the amplitude of the olfactory nerve evoked field potential that was recorded in the olfactory bulb. By contrast, the lidocaine block did not significantly alter the spontaneous activity of single units in the bulb, nor did it alter the field potential evoked by electrical stimulation of the lateral olfactory tract. Lidocaine block also did not change the temporal patters of action potential or their synchronization with respiration.

**Conclusions:**

Spontaneous activity in neurons of the main olfactory bulb is not driven mainly by activity in olfactory receptor neurons despite the extensive convergence onto mitral and tufted cells. These results suggest that spontaneous activity of mitral and tufted is either an inherent property of these cells or is driven by centrifugal inputs to the bulb.

## Introduction

Spontaneous activity in a sensory pathway is the background activity (considered noise by some) against which a sensory response must be detected (e.g. [1]). Spontaneous activity thus contributes to the signal to noise ratio. Spontaneous activity can also increase the dynamic range of a sensory system, since the sensation can be represented as either an increase or decrease in neuronal activity in the sensory pathway (e.g. [2]). In olfaction, recent theories of signal detection have emphasized the importance of action potential (spike) latencies relative to the onset of a sniff cycle (e.g. [3]). However, such analyses do not consider the presence and/or timing of spontaneous action potentials.

Spontaneous activity also has received considerable attention because of its probable role in the formation, maintenance and possible modulation of synaptic interactions in the central nervous system. For example, Katz & Shatz [4] highlighted the importance of spontaneous activity in the development of thalamic synaptic connections in the visual pathway. More recently, Yamada et al. [5] demonstrated that both pre and postsynaptic spontaneous neural firing markedly affected axon branching during development in co-culture preparations of thalamus and cerebral cortex where synaptic connections between the two cell types form *in vitro*. In the olfactory system recent evidence suggests that both presynaptic [6], [7] and postsynaptic [8] activity may be required for the establishment and/or maintenance of a normal olfactory sensory map. Yet the source(s) of spontaneous activity in a given neuron in the central nervous system is largely unknown. This paper explores the source(s) of spontaneous activity in neurons of the main olfactory bulb (MOB).

The principle output neurons of the MOB, mitral and tufted cells, receive excitatory input from olfactory receptor neurons (ORNs) [9], [10]. Based on the numbers of ORNs and the numbers of mitral and tufted cells in rodents, it has been estimated that 100–200 ORNs converge onto each mitral or tufted cell [11]. ORNs themselves are spontaneously active and in anesthetized rats this activity ranges from 0.3 to 11.5 Hz (e.g. [12]). Mitral and tufted cells are also spontaneously active in anesthetized rats with means of 14.7 and 16.6 Hz, respectively [13], [14] (but also see [15]). Given the convergence of ORNs onto mitral and tufted cells, it is possible that the spontaneous activity in ORNs could drive much of the spontaneous activity in mitral and tufted cells via excitatory synapses. The primary aim of this paper is to test this hypothesis by examining the effects of blocking action potential conduction in the olfactory nerve (ON) with lidocaine.

## Results

**Figure 1:**
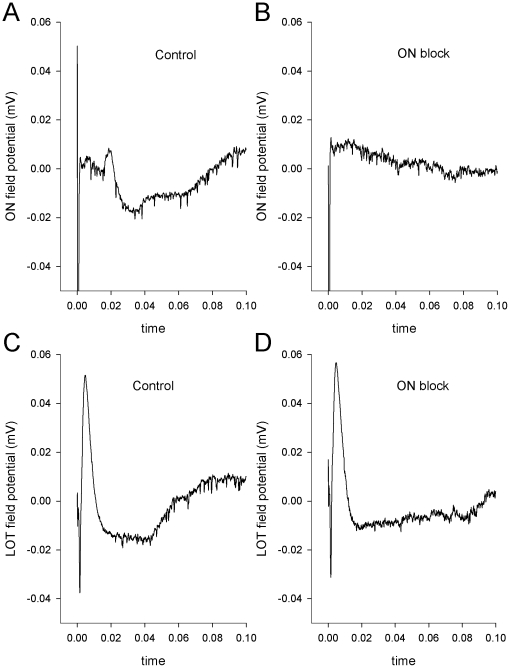
The effects of olfactory nerve block on field potentials. Field potentials recorded in the main olfactory bulb were evoked by stimulating the olfactory nerve (ON field potential, A and B) and the lateral olfactory tract (LOT field potential, C and D) before (control, A and C) and after (ON block, B and D) applying lidocaine to the olfactory nerve.

The main results presented below are based on MOB recordings from 29 anesthetized, freely breathing Sprague-Dawley rats. To evaluate the effects of blocking the ON, it was first necessary to demonstrate that application of lidocaine to the ON would block the conduction of action potentials from the nasal epithelium to the MOB in a substantial fraction of ORN axons. The first experiment demonstrates that lidocaine blocks responses recorded in the MOB evoked by electrical stimulation of ORN axons. Control ON stimulation evoked a field potential in the MOB (Fig. 1A). Figure 1B shows an example where the ON-evoked field potential was completely blocked by lidocaine. Similar results were obtained regardless of where the field potential was recorded (more rostral, more caudal, more medial, more lateral, superficial, or deep), though the waveform of the field potential changes with depth [16].

**Figure 2:**
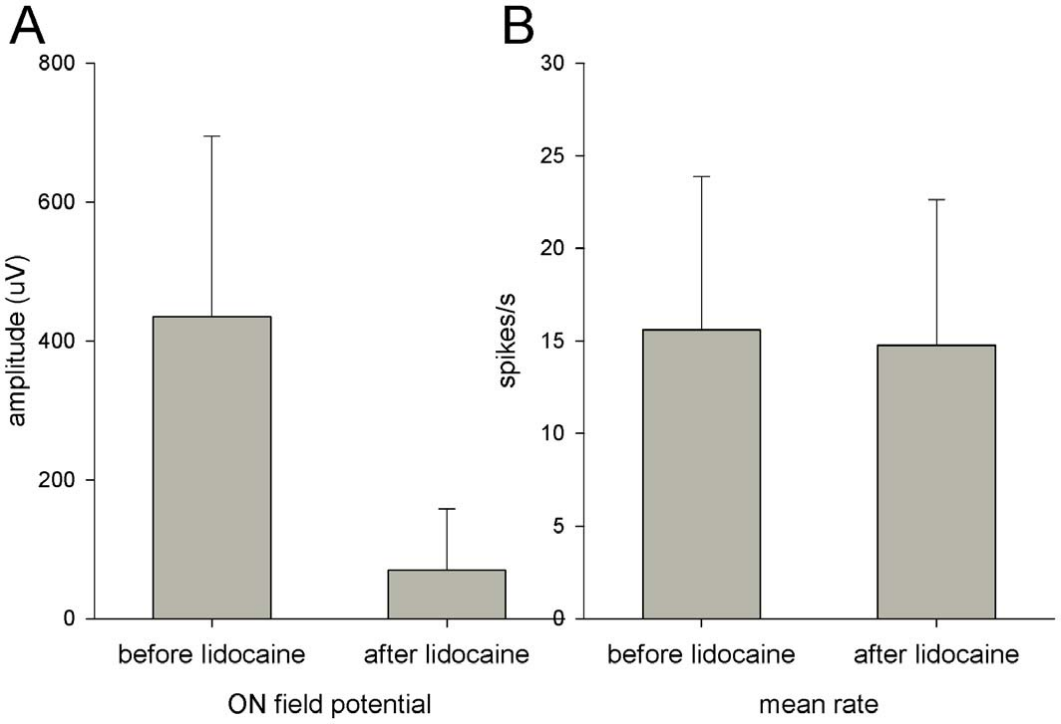
The effects of olfactory nerve block on mean rate. The mean change in the amplitude of the ON-evoked field potential (A) caused by lidocaine application is compared to the mean change in the rate of spontaneous activity (B).

To control for a direct effect of lidocaine on the olfactory bulb, in many experiments field potentials were also evoked by electrical stimulation of the lateral olfactory tract (LOT) under control and lidocaine treatment conditions. As shown in Figs. 1C and 1D, the LOT-evoked field potential was relatively unaffected by application of lidocaine to the ON. Table 1 displays field potential measurements for each experiment. Figure 2A shows the mean changes in the ON-evoked field potentials.

**Table 1:** Effects of ON block on MOB units.

Unit	layer	control epi FP (uV)	epi FP after block (uV)	control LOT FP (uV)	LOT FP after block (uV)	Control rate (Hz)	control SD	rate after block (Hz)	SD after block
040507A	epl	290	85			17.1	6.8	16.8	6.6
041207E	gr	372	14			6.1	2.8	5.4	3.1
041707A	epl	140	0	510	565	19.0	5.4	19.1	4.9
041707E	gr	380	0	900	870	10.7	5.2	10.7	5.2
041707B, C	mcl	340	0	370	350	5.0	1.7	5.3	2.1
042407D,E	epl	300	50	700	612	11.9	5.1	11.5	5.1
042407H	gr	600	0	2300	2300	1.4	0.0	1.1	0.1
042407I	gr	400	45	360	400	3.5	1.8	2.6	2.0
050307F	epl	240	0	712	737	12.8	3.3	15.8	5.7
050307G	epl	130	40	550	562	10.4	3.9	12.5	4.5
050307D	glom	375	105	412	444	25.9	2.9	24.3	1.9
050307J	mcl	170	5.5	290	235	10.4	1.9	11.6	2.1
050807F	mcl	325	0	675	650	24.4	3.7	22.6	4.6
051507G	gr	762	0	925	962	8.2	2.4	8.3	2.0
051507D	mcl	215	30	260	260	23.3	10.8	24.4	13.1
051507E,F	mcl	535	225	1000	488	22.7	6.0	20.7	5.4
051707C	epl	325	0	827	962	23.6	6.6	20.5	6.4
051707F	epl	800	250	190	310	33.1	7.1	34.5	7.0
051707H	epl	550	260			18.0	3.5	14.4	3.6
061907A	gr	375	120			32.0	5.9	28.5	6.3
071007E	epl	405	235			18.0	11.6	11.8	4.9
071007G	epl	290	190			11.8	1.8	12.8	2.8
071207D	glom	585	25			13.4	2.0	11.9	1.8
071207E,F	glom	380	45			13.0	3.0	11.6	2.8
071907C	glom	1375	87	350	450	19.3	4.6	13.4	9.7
071907A,B	mcl	646	0	775	837	10.2	7.9	11.9	8.5

**Figure 3:**
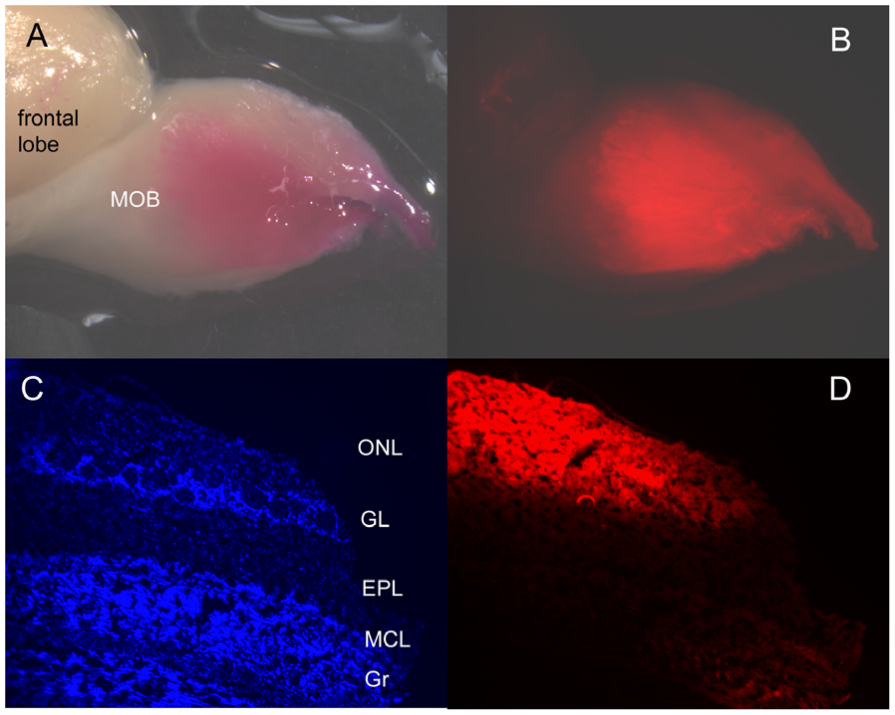
Histological evaluation of the extent of the olfactory nerve block. DiI was applied to the cut ends of the exposed olfactory nerve. (A) shows a whole mount, bright field view of the main olfactory bulb. (B) shows the same view as A under fluorescence. (C) shows a section through the olfactory bulb showing the cell bodies with DAPI staining; the olfactory nerve layer (ONL), the glomerular (GL), external plexiform (EPL) and mitral cell (MCL) and granule cell (Gr) layers of the bulb are indicated. (D) shows the DiI staining in the same section.

To estimate the fraction of olfactory nerve axons blocked during lidocaine application, in 2 anesthetized animals the ON was surgically exposed as in a lidocaine experiment and then the exposed axons were cut. DiI was applied to the cut axons, the animal was perfused with 10% formalin, decapitated, and the head stored in a jar for 14 months. This was sufficient time for the DiI to be slowly transported through the axonal membranes to the MOB; in each animal DiI was applied to both the right and left ONs. Figures 3A and 3B shows photographs of the DiI stain in one whole bulb. About 30% of the bulb's surface is stained, and this was similar for all 4 bulbs. Figures 3C and 3D show that the DiI staining was restricted to the nerve layer of the bulb. Similar results were obtained from each bulb of the 2 rats. In preliminary experiments, the DiI was applied to the cut axons of 2 animals for about 11 hours each while the animals was maintained under anesthesia, but this was not enough time for the DiI to be transported to the bulb and no DiI staining was observed there.

Once we were able to demonstrate the lidocaine block of the ON, we tested how blocking ON activity altered MOB neuron spontaneous activity. The effects of lidocaine ON block on spontaneous activity of bulbar neurons was evaluated for 26 single units (Table 1). After each unit was isolated, control ON- and LOT-evoked field potentials were recorded and measured. Then, the lidocaine block was applied and the ON- and LOT-evoked field potentials were re-recorded. Changes in the field potential amplitudes and the spontaneous activity were evaluated. The mean control ON-evoked field potential significantly decreased from 435 +/- 260 to 70 +/- 89 µV (t = 1×10^−7^, paired t-test) as a result of the lidocaine block to the olfactory nerve (Fig. 2A). By contrast, the LOT-evoked field potential did not change significantly (673 +/- 476 µV in control versus 666 +/- 468 µV with ON lidocaine block; t = 0.9, paired t-test). For 9 units, epithelial lidocaine abolished the ON-evoked field potential whereas the LOT-evoked field potential did not change significantly in those units (t = 0.3, paired t-test).

In contrast to the significant decrease in the ON-evoked field potential, there was no significant change in the mean spontaneous activity of the 26 units (15.6 +/- 8.3 spikes/s in control versus 14.8 +/- 7.9 spikes/s during lidocaine block of the ON, t = 0.15, paired t-test and Fig. 2B). When data for each unit was normalized as the percent change in the rate, the mean percent change was 4.6% which was not statistically significant (p = 1.07).

There was also no obvious difference in the temporal pattern of action potentials between the spontaneous activity in control versus after lidocaine ON block using the coefficient of variation (CV) as a measure. Units that fire action potentials more regularly have a lower CV than units that exhibit a more bursting pattern. There was no significant difference between the CV in control versus lidocaine (0.30 +/- 0.17 versus 0.34 +/- 0.19; t = 0.2, paired t test). There was also no obvious change in the interspike interval histograms before and after lidocaine block and therefore autocorrelations were not done.

In most of the units recorded, respiration was monitored and recorded and in 67% of these units the spontaneous activity was synchronized with respiration. Lidocaine application to the ON did not alter this pattern. Lidocaine block also did not cause units whose activity was not synchronized to become so. In a few units, the spontaneous activity oscillated with a period of several minutes [17]. In most cases, ON block did not alter the amplitude or frequency of these slow oscillations. However, in one unit, ON block reversibly reduced the slow oscillations and in another slow oscillations began after lidocaine application.

Lastly, there was no obvious pattern suggesting that any subclass of bulbar neurons changed its spontaneous activity after the ON was blocked. Based on a dye spot confirmation of the recording site, 6 units were from the mitral cell body layer, 4 from the granule cell layer, and 4 from the glomerular layer. Two of the units recorded in the mitral cell layer could be antidromically activated by LOT stimulation. Based on the polarity of the field potential and recording depth, 10 additional units were from the external plexiform layer (EPL), and 2 additional units were from the granule cell layer. Four of the units recorded in the EPL could be antidromically activated by LOT stimulation. These units are likely tufted cells.

## Discussion

This study provides evidence that the spontaneous activity of neurons in the MOB is not driven primarily by spontaneous activity in ORN's despite the 200 to 1 convergence of ORNs onto mitral and tufted cells. Spontaneous activity in MOB neurons did not change significantly when conduction in the ON was blocked by lidocaine. The decrease in the epithelial-evoked field potential was used as a measure of ON block. One indication that the lidocaine blocked a large number of ON axons is that the field potential could be blocked regardless of where in the bulb the field potential was recorded. A second indication was that the field potential was completely abolished in many applications.

A histological assessment of the number of ON axons blocked was obtained by DiI staining of ON axons from the same part of the ON where the lidocaine was applied. DiI is a hydrophobic and lipophilic cyanine dye that is retained in the lipid bilayer of membranes. The DiI slowly moved from the cut ends of these axons to the MOB where it was restricted to the nerve layer of the bulb (Fig. 3D). The amount of DiI stain is likely an underestimate of the number of axons that were exposed to lidocaine since not every axon would be expected to take up the dye and/or transport it to the MOB. Lidocaine, by contrast would be expected to easily diffuse to most, if not all, of the axons in the exposed region of the ON.

We conclude that lidocaine blocked a large number of ORN axons and that if ORN input to the MOB was a major contributor to spontaneous activity in the bulb, lidocaine application would have caused a measurable change. Direct ORN input to mitral and tufted cells is excitatory [9], [10], [18], and if this excitation contributed to the spontaneous activity in these cells, blocking this input should have decreased spontaneous activity. On the other hand ORN input also activates inhibitory interneurons that in turn synapse on mitral and tufted cells [19]. If these circuits contributed to spontaneous activity, blocking this pathway would have increased spontaneous activity. This would apply to inhibitory juxtaglomerular cell and/or to granule cell circuits. In fact, the lidocaine ON block caused neither an increase nor a decrease in units from the mitral, external plexiform and glomerular layers. Nonetheless, we cannot exclude the possibility that the spontaneous activity of a small percentage of bulbar neurons is influenced by ORN input. In frog, partial surgical deafferentation of mitral cells significantly increased spontaneous activity [7] suggesting a tonic inhibition of mitral cells in frog via inhibitory interneuron circuits. These inhibitory circuits in frog are tonically-driven by ORN spontaneous activity.

The spontaneous activity of bulbar neurons recorded in early mammalian slice preparations was much lower than *in vivo* preparations (e.g. [20]). More recently, higher levels of spontaneous activity have been observed *in vitro*. For example, Hayar et al. [21] estimated that external tufted cells fire about 15 spikes/s, an average of 5.4 spikes per burst and 3.3 bursts per second. Hamilton et al. [22] report the spontaneous activity of tufted cells in the external plexiform layer as 31 Hz in mouse. Palouzier-Paulignan et al. [23] recorded mitral cells with spontaneous activity up to 13 Hz. These values are similar to the rates reported in this report. An obvious difference between *in vivo* and *in vitro* preparations is the absence of the olfactory epithelium and functional inputs for ORNs. Thus, the similarity between spontaneous activities of *in vivo* and *in vitro* recordings is consistent with the hypothesis that input from ORNs do not contribute significantly to the spontaneous activity of bulbar neurons.

The similarity between spontaneous activities of *in vivo* and *in vitro* recordings also suggests that spontaneous activity is an intrinsic property of the MOB. Indeed, spontaneous activity is a property of some cultured bulbar neurons such as a population of dopaminergic periglomerular cells [24]. External tufted (ET) cells recorded in slice preparations generate spontaneous rhythmical bursts of action potentials [21]. Paired recordings in bulb slices showed that slow population bursts in mitrals cells were synchronized with the spontaneous discharges in ET cells [25].

The entrainment of spontaneous activity with respiration that was observed in some bulbar neurons was not altered by blocking the ON. Previous studies to investigate the mechanism(s) by which such spontaneous activity is synchronized with respiration are contradictory [26]. Modulation of mitral/tufted unit activity by respiration persisted when animals were tracheotomized [27] suggesting that airflow past the olfactory epithelium is not required. On the other hand, mitral/tufted cell activity was uncoupled from respiration by naris closure [28] though the activity was also greatly attenuated. More recently, Grosmaitre et al. ([29]) demonstrated that olfactory sensory neurons can respond to mechanical stimulation and they suggest that this factor may provide a driving force to synchronize bulbar activity with breathing cycles. Respiratory synchronization could persist after ON block if rhythmic activity is an inherent property of the MOB and its synchronization requires few active inputs from the nasal epithelium [30]. As indicated above, neurons such as ET cells generate rhythmic burst in the slice preparation [21]. In the present study, lidocaine probably did not block all input from the nasal epithelium and it is possible that spontaneous action potentials in the unblocked axons was sufficient to entrain bulbar activity to respiration.

In both rat and frog, GABA antagonists affect spontaneous activity indicating that local inhibitory circuits and/or inhibitory centrifugal inputs to the MOB modulate mitral cell activity [31], [32], [33]. Although the role(s) of centrifugal input to the MOB has been investigated [34], [35], [36], [37], the effect of centrifugal input on spontaneous activity in the MOB has not been studied. Subsequent experiments will explore the role of centrifugal inputs in modulating bulbar spontaneous activity.

## Materials and Methods

### Anesthesia and surgical procedures

#### Animals

This study was carried out in strict accordance with the recommendations in the Guide for the Care and Use of Laboratory Animals of the National Institutes of Health. All procedures also adhered to guidelines established by the American Association for the Accreditation of Laboratory Animal Care and were approved by the University of Cincinnati Institutional Animal Care and Use Committee (Protocol No. 10-05-20-01, Principal investigatory Edwin Griff). All efforts were made to minimize animal suffering and to reduce the number of animals used. Experiments were performed on adult male Sprague-Dawley rats weighing between 313 and 457 g. Animals were anesthetized with 4% chloral hydrate, ip. The plane of anesthesia was maintained with additional ip chloral hydrate to be deep enough so that a pinch of the paw did not cause a withdrawal reflex, but did desynchronize the EEG [38]. All animals breathed freely; they were not tracheotomized. Respiration was monitored and recorded by a displacement transducer in contact with the wall of the abdomen. Synchronization of spike activity with the respiratory cycle was determined by constructing histograms triggered by the beginning of inspiration. A heating pad maintained body temperature at 35–37°C.

The cortical EEG was recorded with a bipolar electrode made from 250 um stainless steel wires, insulated except for the cut tips with one electrode about 500 um longer than the other. A small (3 mm diameter) area of the parietal cortex was exposed by thinning and removing the skull and the dura. The electrode was inserted in the cortex with the shorter electrode about 200 um deep and secured to a skull screw with dental acrylic. The EEG was recorded with a differential amplifier (Thornton Associates, 400, 410, 418), displayed on an oscilloscope (Tektronix, model 5111A), digitized (CED, Micro 1401), and recorded on a computer using CED Spike2 software.

#### Surgery

Blunt dissection was used to expose the orbit, dorsal surface of the skull and lateral surface of the temporal bone. The muscles of the orbit and several that attach to the temporal bone were dissected, the temporal process of the zygomatic bone and the zygomatic process of the temporal bone cut, and the eye removed. The bone covering the dorsal surface of MOB was thinned and removed. The bone covering the lateral surface of the LOT was also removed. The dura was then removed and the surfaces kept moist with saline. In addition, bone over the dorsal and dorso-lateral surfaces of the olfactory nerve rostral to the cribiform plate was exposed and the area was kept moist with saline.

### Isolation and identification of MOB units

#### Stimulation and recording electrodes

A recording micropipette was positioned in the MOB, and two bipolar stimulation electrodes also were positioned. One stimulation electrode contacted the axons of the olfactory nerve at the rostral extreme of the exposed area. This “ON electrode” evoked a field potential in the MOB that has a prominent slow negative component, which reverses polarity when the recording electrode passes through the mitral cell body layer (MCL). The other stimulation electrode contacted the LOT and produced antidromic action potentials in the axons of tufted and mitral cells. Stimulation of the “LOT electrode” also produced a field potential in the MOB.

The LOT and ON stimulation electrodes were constructed from 250 um stainless steel wire insulated except where it was bent and exposed to make contact with the nerve or tract. Square-wave pulses of constant current, typically 50 uS, 200–400 uA, were applied using Grass stimulators (model S44), stimulation isolation units (SIU5) with 100 kOhm resistors in series to ensure that the currents was minimally affected by variability in tissue resistance or electrode contact from animal to animal. The stimulator was triggered manually or at a constant rate (usually 0.5 Hz). Recording pipettes were pulled from thin-walled borosilicate glass capillaries (1.50 mm O.D., 1.12 mm I. D.) on a Brown-Flaming puller (Model P-80) and filled with 2% pontamine blue dissolved in 0.5 M NaCl solution.

#### Isolating cells

Single units were isolated by driving the recording electrode through the MOB in 5 - 10 um steps (Kopf hydraulic micropositioner, model 650) while watching and/or listening for action potentials. Action potentials were amplified and filtered (Dagan, 2400A amplifier), observed on a storage oscilloscope (Tektronix, 5111) and listened to on an audio monitor (Toshiba RT-S801). Once a unit was detected, the electrode was positioned to maximize the spike amplitude. The criterion for a single unit was constant amplitude and waveform of the action potentials and an absolute refractory period of at least 2 ms. The large spikes of a unit were detected with a window discriminator (Dagan, WD-2), digitized (CED, Micro 1401), and recorded on a computer using CED Spike2 software; data was also recorded on VCR tape (Medical Systems, PCM4/8). Units with large irregular oscillations of spontaneous activity were not used. In some cases spike sorting CED Spike2 software was used to ensure that each action potential of a single unit was recorded.

Units were identified by recording depth, the polarity of the field potential, antidromic activation, and/or a dye spot. Dye deposited from the tip of the recording pipette marked the recording site by passing cycling current (-10 uA, 7 sec on, 3 sec off) through the electrode for 10 min. At the end of the experiment, the animal was perfused with phosphate-buffered saline (pH 7.4) followed by 10% buffered formalin via a cardiac puncture to the ascending aorta. Following perfusion with about 300 ml of formalin, the brain was removed and stored first in 10% formalin, and then 10% formalin with 20% sucrose added. The olfactory bulbs were frozen (ethanol and dry ice) and sectioned using a sliding microtome. Sections with a blue spot were mounted, stained with neutral red, dehydrated in alcohol, substituted with xylene and coverslipped. The position of the intense blue spot indicated the recording site.

Recording depth was estimated from the displacement of the microelectrode from the bulb surface using the display of the Kopf micropositioner. As the microelectrode was advanced, field potentials were evoked from stimulation of the ON or LOT. The large slower wave of the field potential is negative at the surface and reverses polarity at the mitral cell body layer [16]. When a unit was isolated, we attempted to antidromically activate the cell from the LOT. Antidromic activation was indicated by an action potential similar in amplitude and shape to a spontaneous action potential that could be evoked at a constant short latency when threshold was reached.

Spontaneous activity was recorded in control conditions before lidocaine application (see below) and usually before recording control ON- and LOT-evoked field potentials. The sampling time varied from 22 to 252 s with a mean of 76.3 +/- 54.7 s; longer sampling times were employed when the activity not as steady. Spontaneous activity was again recorded after lidocaine application and after recording field potentials in the lidocaine condition to ascertain that conduction in the ON had in fact been blocked. The sampling time in lidocaine varied from 24–390 s with a mean of 93.6 +/- 73.2 s.

### Olfactory nerve block

#### Lidocaine

A small piece of cotton saturated with 2% lidocaine HCl (Hospira, Inc.) was used to apply the lidocaine to the ON caudal to the epithelial stimulation electrode. ON block was assessed by monitoring the decrease in amplitude of the ON-evoked field potential after lidocaine application. A maximal block was usually obtained in less than 10 min. There was no decrease in the field potential after application of cotton saturated with saline. After recording the spontaneous activity of a unit during ON block, the lidocaine was removed by several flushes of saline. The epithelial-evoked field potential usually recovered, at least partially, within 20 minutes.

#### Cold

In early experiments, cooling the ON caudal to the ON stimulation electrode was used to reversibly block ON conduction. A copper probe that could be cooled by a Peltier device was positioned in contact with the ON caudal to the epithelial stimulation electrode. Cooling the probe to 33–35°C, reversibly blocked ON conduction, as indicated by a decrease in the amplitude of the epithelial-evoked field potential.

Cold block of the ON caused a decrease in spontaneous activity in many units. However, for some of these unit recordings where cold had decreased activity, after recovery from cold, the ON was severed. Cutting the ON did not cause a similar decrease in activity. We therefore suspected that the decrease in activity from cold was some sort of artifact. Thus, the cold block technique was abandoned and replaced by the lidocaine technique. Cold could have affected the MOB directly, though there was no change in the surface temperature or could have altered blood flow to the MOB.
